# Editorial: Analysing Psychosocial and Contextual Factors Underpinning Bullying and Cyberbullying

**DOI:** 10.3389/fpsyg.2019.02602

**Published:** 2019-11-19

**Authors:** Eva M. Romera, Rosario Ortega-Ruiz, Grace Skrzypiec, Rita Zukauskiene

**Affiliations:** ^1^Universidad de Córdoba, Córdoba, Spain; ^2^Flinders University, Adelaide, SA, Australia; ^3^Mykolas Romeris University, Vilnius, Lithuania

**Keywords:** bullying, cyberbullying, risk and protective factors, psychosocial, context variables

Studies about bullying have identified it as a public health problem, with serious academic and psychosocial consequences. The extant literature defines bullying as an intentional phenomenon, repeated over time, which is sustained by the relational dominion-submission model established between victims and aggressors, and that is generally maintained by a lack of bystander intervention and indifferent bystander attitudes. This behavior pattern of aggressive interaction has been further broadened and diversified through the use of information and communication technologies (ICT) that has given rise to what is commonly known as cyberbullying.

Although the multicausal nature and negative effects on the well-being of those involved in bullying and cyberbullying have been clearly identified, high levels of involvement in such negative behavior remain. In the study of bullying and cyberbullying, the implication to cognitive, social, and moral competencies, which young people display in interpersonal situations, and which denote aspects of their social personality, have also been examined. Moreover, contextual dimensions, such as social status, group norms, and school climate have been identified as elements associated with the risk of school children becoming victims or aggressors and with the possibility of such behavior reoccurring.

## Individual Risk and Protective Factors

Although bullying and cyberbullying have been widely studied throughout the past decades, there are still many gaps in our knowledge regarding individual and contextual factors that could potentially be the cause or consequence of this aggressive behavior (Zych et al., 2018). The objective of this Research Topic is to advance many of the research areas that still need to be addressed.

More than half the studies published in this Research Topic have paid attention to individual characteristics as explanations for involvement in bullying and cyberbullying. Predominantly moral beliefs and values have been underscored as important. Four studies have highlighted the moral domain as a relevant variable in understanding involvement in peer violence. In a 1-year longitudinal study with 1,250 Swedish children from fourth to fifth grade, Thornberg et al. have contributed to this Research Topic using multilevel analyses that have shown that children with little individual moral disengagement, as well as strong defender self-efficacy, are less inclined to be involved in bullying perpetration. A comparable analysis of 2,000 adolescents from Italy and Greece by Lazuras et al. found that engagement in cyberbullying was associated with moral disengagement, while hierarchical linear regression analyses revealed cross-national differences in the mediating variables of this relationship. Examining data from 1912 Spanish students aged 14 to 18 years, the role of moral disengagement was shown by Cuadrado-Gordillo and Fernández-Antelo, to mediate the perception of cyberbullying and involvement in cybervictimization. Finally, Romera et al. used stick-figure cartoons representing bullying with 1,150 schoolchildren between the ages of 6 and 11 years and showed that school children interpret and evaluate aggressive bullying behavior as a moral transgression associated with emotions such as guilt, shame, and indifference. This study made it clear that moral attributions of the phenomenon depend on one's perspective, especially when children have had an experience of being involved as victim or aggressor.

Social motivation has also been recognized as a significant factor associated with bullying and cyberbullying. Cognitive representations about the desired outcome in social interactions help us to understand why some children and adolescents are involved in risky behavior (Romera et al., 2017). In this research domain, Graf et al. used structural equation modeling with data collected from Austrian students to show that sensation seeking is an important element for explaining cyberbullying.

Along with other variables, Internet use was identified as a risk factor for bullying, cyberbullying and cyberhate by Blaya and Audrin who studied 1,889 young French individuals aged 12–20 years. Structural Equation models in their study showed that cyberhate perpetration was related to time spent online, victimization, belonging to a deviant youth group, as well as having positive attitudes toward violence and racism. A multiple mediation analysis further suggested that trust in institutions diminished the tendency to perpetrate hateful aggression. Meanwhile, Felipe-Castaño et al. analyzed data from 1,108 university students and showed that Internet use and the presence of psychopathological symptomatology varied according to the intensity of the cyberbullying and cyberaggression.

Furthermore, gratitude and physical activity were identified as protective factors by Rey et al.. With data from a total of 1,617 Spanish adolescents, Rey et al. showed that gratitude moderated the relationship between bullying victimization and suicide risk, particularly in girls. Additionally, among 1,248 adolescents aged 11 to 18 years, Méndez et al. showed that the practice of non-competitive physical activity is related to low involvement in aggression and deviant behavior.

## Contextual Risk and Protective Factors

On another level, contextual factors have been shown to be essential in the prevention of aggression and victimization, as research has confirmed the influence of the peer group on the perceptions, attitudes and behavior of children and adolescents (Salmivalli, 2010). The social and developmental mechanisms associated with peer influence are paramount for understanding bullying for two main reasons. Firstly, the influence is social because bullying is identified as a group phenomenon in which a large number of people are directly and indirectly involved. Secondly, the influence is developmental because the peer group exists for children and adolescents in one of the most influential contexts of their development and learning.

In this Research Topic different studies have explored the role of the peer group. Garandeau et al. used multilevel regression analysis with data from ~3,000 students from Netherlands and Austria to show that classroom size was negatively related to bullying. The association was mainly due to differences in the level of popularity of victims and aggressors in large and small classes. Likewise, the socio-ecological aspects of children's lives were examined by Foody et al.. With data from 2,400 Irish 12 to 15 year old students, and using regression models, they showed that friendship quality within the context of cyberbullying might be highly influential in determining psychological well-being. Meanwhile examining data from 3,407 primary school students, Moyano et al. showed that low negative relationships and the sense of social integration mediated the relationship between the type of school and involvement in different forms of bullying—particularly relational bullying.

Scientific advances have also explored the influential role of adults, parents and teachers, in children's social development. For example, parenting styles have been widely studied in association with involvement in bullying (Gómez-Ortiz et al., 2015), and cyberbullying (Gómez-Ortiz et al., 2018). The studies presented in this Research Topic advance the research field concerning the role of parents and teachers. Brighi et al. using structural equation modeling with data obtained from 3,602 students from Italian Secondary Schools found that low levels of parental monitoring and negative emotional symptoms were risk factors for cyberbullying perpetration and problematic internet use. Cybervictimization was also examined by Álvarez-García et al.. Using path analysis with a sample of 3,360 Spanish adolescents aged 11 to 18 they found that parental control had an indirect protective effect on being involved in cyber-victimization, and this was mediated by impulsivity and high-risk internet behavior. Path analysis was also used by De Luca et al. who studied 120 teachers and 1,056 students of secondary school. They showed that teachers who perceived themselves as more competent and who were satisfied with their job, were more prone to intervene in cases of bullying and victimization.

## Cross-Cultural Comparisons

Cross-cultural comparisons in the level of bullying and associated variables were undertaken by Rodríguez-Hidalgo et al. and by Samara et al. with large samples of adolescents in two separate studies. Using multiple linear regression analysis, Rodríguez-Hidalgo et al.. highlighted that ethnic-cultural victimization and aggression were associated with bullying aggression and victimization.

In their study, Samara et al. examined data from a total of 3,186 school children aged 12–15 years from four countries (Israel, Palestine, Germany, and Greece) and five different ethnic groups. This large cross-ethnic and cross-country study showed that bullying and victimization rates can only be compared if structural equivalence and structural isomorphism are validated. Results of this research evidenced that students may perceive the meaning of bullying in a different way, so comparisons between rates are not valid.

The meaning of bullying, the way it is measured and the way that children and adolescents understand it has been debated and analyzed in previous studies with quantitative data (e.g., Skrzypiec et al., 2018). In this Research Topic, O'Brien reviewed qualitative studies and showed that young people have different perceptions of bullying, both in the understanding of the behavior as well as the impact it has on individuals. Furthermore, this study alluded to the complexity and interexchange of roles that may occur within bullying.

## Implications of the Findings

Studies in this Research Topic advance our knowledge of the interactive dynamics of psychological and contextual factors and their relationship to bullying and cyberbullying. The focus has included individual and contextual characteristics, particularly how these are understood either as risk factors or as consequences of student involvement in bullying and cyberbullying. Through diverse methodological designs, studies in this Research Topic have presented evidence that psychosocial, moral, motivational and emotional elements, and the peer and adult contexts are important for understanding this complex behavior. The studies highlight that understanding bullying and cyberbullying requires that we globally address the different factors of influence identified by the scientific literature. The manuscripts included in this Research Topic update the body of knowledge that provides relevant information for identifying conditions associated with bullying and cyberbullying and that interfere with the safety of children at school.

The results provided through the studies in this Research Topic are useful for designing educational cross-cultural programs aimed at preventing bullying and cyberbullying. Strengthening the social structure of the peer group would allow teachers, practitioners, and policymakers to improve social relationships among their students and in doing so prevent such damaging phenomena.

## Author Contributions

All authors listed have made a substantial, direct and intellectual contribution to the work, and approved it for publication.

### Conflict of Interest

The authors declare that the research was conducted in the absence of any commercial or financial relationships that could be construed as a potential conflict of interest.
